# Pregnancy: Its Influence on the Development and Progress of Pulmonary Tuberculosis

**Published:** 1863-01

**Authors:** A. P. Dutcher

**Affiliations:** Enon Valley, Pennsylvania


### PREGNANCY:

### ITS INFLUENCE ON THE DEVELOPMENT AND PROGRESS OF PULMONARY TUBERCULOSIS.

By A. P. DUTCHER, M. D., Enon Valley, Pennsylvania.

This is a question of commanding interest. From our reading and acquaintance with physicians, we are satisfied that it has never received that attention which its importance demands. Medical writers are quite at variance in their opinions on the subject. The greatest confusion prevails on every print connected with it; and from our studies, we have found it impossible to harmonize the different and conflicting opinions expressed with the facts which preside in the case. This will appear quite evident, if I call your attention for a few moments to some of these opinions as they stand recorded on the pages of our most prominent medical authors. For brevity, we will not take you back farther than the last edition of Morton's *Illustrated Pulmonary Consumption*, published in 1839.

On page 206, he says: "The duration of phthisis is greatly modified by the peculiar functions of the female constitution. During the period of pregnancy the morbid action is suspended in the lungs, while all the resources of the system are devoted to the uterine functions. Lactation produces in degree the same effect; and it is thus that child-bearing women, although decidedly consumptive, enjoy a state of comparative health for many years; but the disease is only latent, and prone to recur with fatal violence when this check is removed."

Dr. Montgomery, in his work, *Signs of Pregnancy*, when speaking of the sanitary influence of pregnancy upon co-existing disease, says (p. 25): Indeed, I think we have sufficient evidence to justify the belief that pregnancy acts in a good degree as a protection against the reception of disease, and apparently on the common principle, that during the continuance of any one very active operation in the system, it is less to be invaded or acted upon by another; thus it has been observed that during epidemics of different kinds, a much smaller portion of pregnant women have been attacked than others; and when women have been laboring under certain forms of disease, happen to conceive, the morbid affection previously existing is greatly mitigated or suspended for the time, as has been frequently observed in phthisis."

Dr. Edward Warren, in his *Fisk Fund Prize Essay*, on the influence of pregnancy in developing pulmonary tuberculosis, published in the *American Journal of the Medical Sciences*, for July, 1857, contends, with much ingenuity and no little learning, that pregnancy is antagonistic to the development of pulmonary tuberculosis, and that when it occurs during the progress of the disease, it is opposed to the continuation of the tubercular diathesis, and may in this way contribute to the arrest of the local lesion. That such results are due to pregnancy, he thinks is evident from the following considerations:

"1. Pregnancy produces a condition antagonistic in the economy.

"2. Pregnancy is a vital process, a highly physiological act, and hence its existence is incompatible with the progress and perfection of a purely morbid effort.

"3. Pregnancy diverts the forces and fluids from the lungs, and to the uterus.

"4. Pregnancy is regarded by a large majority of medical men as antagonistic to the march of phthisis.

"5. Pregnancy depends upon the existence of certain susceptibilities which are inherent in the female system, and hence it is more universal in its operation than any other imaginable cause.

"6. Pregnancy, coition, etc., are particularly desired by women affected with phthisis, which constitutes a *pointing of nature* towards a *remedy* for the evils by which the system has been invaded."

Dr. L. M. Lawson, in his new work, *Phthisis Pulmonalis*, says (p. 306): "My own convictions on this subject have been deduced from personal observations, and, although not in the form of statistics, are, at least to myself, not the least conclusive on that account. It is my conviction, then, that in the tubercular predisposition, or even the *precursory* stage of phthisis, the occurrence of pregnancy, under favorable circumstances, and frequently repeated, so change the vital actions as to delay or entirely arrest the impending local deposit. And what I mean by favorable circumstances is, that the person should be in the enjoyment of a fair degree of general health and strength, the pregnancy progress regularly to its natural termination, and that the subject during the time be placed under proper hygienial conditions in regard to exercise, clothing, diet and habitation." When the disease has become fully established, Dr. Lawson is of the opinion that, if the tubercular deposits be limited, and the patient's strength and digestion good, pregnancy may still retard the progress of the local disease. But if the deposits be extensive, and softening has occurred, gestation will not retard the malady, but, on the contrary, will have a tendency to accelerate it.

Dr. Sweet, in his excellent *Lectures on Diseases of the Chest*, expresses an opinion on this subject quite different from those just given. He says (p. 263): "There is a common impression that pregnancy retards the progress of phthisis. Probably it only renders it latent, and thus an apparent rather than a real advantage is gained. That it produces neither of these results in some cases, I am well convinced; and the practitioner who recommends it to his patient may be disappointed even in a temporary advantage. Even supposing that the progress of tuberculosis is retarded during the existence of pregnancy, what is the final result? As soon as delivery has taken place, the pulmonary diseases usually advances with a greater rapidity, and, in addition, a child with a strong tuberculous tendency is born. Certainly there is no great advantage in these results, and you will, I hope, be disposed to adopt the opinion that I have formed: never to advise pregnancy to a tuberculous female. Cases of this kind will occur often enough, and the evil consequences be experienced, without, or in opposition to our advice."

M. Laus, in his great work, *Pathological Researches on Phthisis*, p. 305, occupies nearly the same ground as Dr. Sweet, although his ideas are not as clearly expressed. "We have," he says, "not been able to decide whether pregnancy is capable of retarding the progress of phthisis; it is evident that numerous facts are required, and several years in a lying-in hospital, before we can have any positive information on the subject. We may observe, however, that perhaps there have been some error and confusion among those who have hitherto admitted such an influence. It is indeed possible that many of the symptoms of phthisis may be less prominent during pregnancy, while the progress of the disease is really unaffected. On the other hand, it is not impossible that after labor the progress may be more rapid than at any previous period; and this difference before and after confinement may, to a certain extent, have given rise to the impression."

MM. Dubreuilh and Grisolle have both presented reports to the French Academy of Medicine, in which they ignore the opinion of antagonism between pregnancy and phthisis, and have attempted to establish the idea that the progress of phthisis is hastened by that particular state. These gentlemen are the only individuals, so far as my knowledge extends, who have made any attempts to furnish us with any statistics on this subject. They have produced forty-eight cases for the solution of this question; but they are limited in number, and too imperfectly described to be of any material use.

We might quote the opinion of several other writers, but they would not add anything to our knowledge on this subject, or assist us materially in its solution. It can not, however, be denied that the weight of authority, as it stands recorded on the pages of medical practice, is in favor of the opinion that pregnancy is antagonistic to the development of phthisis. But when we reflect that this opinion is based exclusively upon theoretical deductions, we do not consider it entitled to very much confidence. In so important a question as this we should have something more than theory; nothing but facts and their legitimate deductions can fill the bill of our wants in this case. Fine-spun theories may do to amuse the man of fanciful intellect, but they are frequently of no value when applied to the wants of suffering humanity. Perhaps some will regard it an unjust criticism, when I say that in some of our most popular medical works theories are taught almost to the exclusion of facts. And in very many of our medical schools the same great error prevails; and hundreds of young men come forth from them annually to enter upon the practical duties of the profession in no way qualified. Hence it is a notorious fact, that not one half of them who graduate ever become successful practitioners. They attempt to reduce their metaphysical vagaries to practice, and the result is almost a uniform failure.

In our profession it is a serious truth that the opinions and theories of great writers and teachers are too often taken for positive knowledge. Antiquated dogmas take the place of the grand truths discovered by the research of modern medical science; facts derived from practical experience and observation must give place to some miserable hypothesis that should never have found a lodgment in the human mind. The young physician thus learns to depend upon others; he adopts their opinions without proper investigation, and he frequently finds, to his great mortification, that they are erroneous and of no practical utility.

If a man would become a successful practitioner of medicine, he must not depend too much on the opinion of others; and, indeed, this is true in every department of active life. If a man would have success, he must stand up for himself. Every man has his own sphere, his own duties, and his own powers to discharge them. This place no other man is to take; his labor no other is to perform. This is the natural order, the divine arrangement. If a man would acquire might of intellect, be a power in the world and gain an immortal name, he must to a great extent travel through the fields of science independently and alone, never adopting a theory or an opinion because it is sanctioned by a great name. And by this we do not mean to intimate that the aid and teachings of others are to be abjured. No, far from it. Let us go to all the great masters of our noble profession, and to all the wisdom which past ages have treasured; of the works of the present time, let us study all those which stand as the indices and prototypes of modern medical science. Let us apply to all the great fountains of knowledge for ourselves, not to fill a reservoir, but to water the plants which grow in our fields. To the young physician I should say, plant your own crops, and reap your own fields, and you will enjoy the sweet fruition of your own labors.

In investigating every subject that relates to the practice of our profession, we should exercise our own judgment, and conscientiously follow its teachings. Especially is this necessary in the case before us, where there is such a contrariety of opinion. If pregnancy is antagonistic to phthisis, its occurrence in a consumptive patient is a fortunate circumstance; if not, it is unfortunate, and he who recommends it is inflicting a great wrong upon his confiding patient. For my own part, I am convinced that pulmonary tuberculosis is not in any way ameliorated by pregnancy.

If pregnancy has such a powerful influence in correcting the tubercular diathesis, and arresting the local lesion, as some authors maintain, we ought at least sometimes to meet with cases of recovery from the fell disease, but such has never been my good fortune, and I have never yet heard of a well authenticated instance in the observation of others. The most strenuous advocates of this theory have never presented a single case of this kind. Dr. Warren, in that elaborate and extensive essay of his, has not furnished a single instance. Dr. Morton mentions one case where phthisis appeared to be suspended, for a long time, by the frequent occurrence of pregnancy, but the individual ultimately succumbed to the pulmonary disorder. Dr. Warren in his essay has labored very diligently to show that pregnancy is a state of plethora, and that this condition is unfavorable to the development of pulmonary tuberculosis. I do not know that this has ever been seriously controverted But that this state will be generally produced in phthisical females by the occurrence of pregnancy, has never been demonstrated. Indeed, experience teaches us to believe the contrary. Pregnancy, for its proper accomplishment, always requires an extra expenditure of vital power, and as phthisis is essentially a disease of weakness and debility, this must inevitably tend to its development in those who have a decided proclivity to the malady. And how often is it the case, that we see young women afflicted with this disorder marry, become pregnant, struggle through it, and fall a prey to the disease within a year. From these considerations, and others that might be named, we are compelled to dissent from the commonly received doctrine, that pregnancy is antagonistic to the development and progress of pulmonary tuberculosis.—*Cin. Lan. & Observer.*

